# Microbial communities in swine lungs and their association with lung lesions

**DOI:** 10.1111/1751-7915.13353

**Published:** 2018-12-17

**Authors:** Tao Huang, Mingpeng Zhang, Xinkai Tong, Jiaqi Chen, Guorong Yan, Shaoming Fang, Yuanmei Guo, Bin Yang, Shijun Xiao, Congying Chen, Lusheng Huang, Huashui Ai

**Affiliations:** ^1^ State Key Laboratory for Swine Genetic Improvement and Production Technology Jiangxi Agricultural University Nanchang 330045 China

## Abstract

Under natural farming, environmental pathogenic microorganisms may invade and affect swine lungs, further resulting in lung lesions. However, few studies on swine lung microbiota and their potential relationship with lung lesions were reported. Here, we sampled 20 pigs from a hybrid herd raised under natural conditions; we recorded a lung‐lesion phenotype and investigated lung microbial communities by sequencing the V3‐V4 region of 16S rRNA gene for each individual. We found reduced microbial diversity but more biomass in the severe‐lesion lungs. *Methylotenera*,* Prevotella*,* Sphingobium* and *Lactobacillus* were the prominent bacteria in the healthy lungs, while *Mycoplasma*,* Ureaplasma*,* Sphingobium*,* Haemophilus* and *Phyllobacterium* were the most abundant microbes in the severe‐lesion lungs. Notably, we identified 64 lung‐lesion‐associated OTUs, of which two classified to *Mycoplasma* were positively associated with lung lesions and 62 showed negative association including thirteen classified to *Prevotella* and six to *Ruminococcus*. Cross‐validation analysis showed that lung microbiota explained 23.7% phenotypic variance of lung lesions, suggesting that lung microbiota had large effects on promoting lung healthy. Furthermore, 22 KEGG pathways correlated with lung lesions were predicted. Altogether, our findings improve the knowledge about swine lung microbial communities and give insights into the relationship between lung microbiota and lung lesions.

## Introduction

To characterize the microbial communities living in or on human bodies and to investigate their roles in human health and disease, the Human Microbiome Project was launched in 2008 (Turnbaugh *et al*., 2007). But this project did not include lung microbiome possibly because people thought there were no bacteria in healthy lungs that time (Beck *et al*., 2012; Dickson *et al*., 2016). With the application of novel culture‐independent methods to microbial detection, more and more studies evidenced the fact that microbiota exists in the lower respiratory tracts (Charlson *et al*., 2011; Erb‐Downward *et al*., 2011). Most respiratory diseases are caused by or accompanied with a bacterial infection (Cilloniz *et al*., 2016), like chronic obstructive pulmonary disease (Marsland and Gollwitzer, 2014), and community‐acquired pneumonia (Rudan *et al*., 2013). Therefore, it is important to reveal the microbial community structure in lung and elucidate its roles in keeping lung healthy and preventing the development of lung disease. However in human, bronchoalveolar lavage samples of healthy participants are difficult to obtain (Martinsen *et al*., 2016). Swine lung could serve as an ideal model for biomedical research of human lung due to its similarity in anatomical size and structure and its easy accessibility (Swindle *et al*., 2012). The microbial samples in swine lungs can be obtained after slaughter, and sample contamination from the oral and upper respiratory tracts can be efficiently avoided.

Moreover, swine respiratory disease caused by microbial pathogens is one of the major diseases in pigs. It is prevalent in modern intensive pig farms worldwide, can cause poor porcine growth performance and in turn leads to serious economic losses to the swine industry (Opriessnig *et al*., 2011). Usually, multiple infectious agents are involved in the development of swine respiratory disease (Brockmeier *et al*., 2002; Maes *et al*., 2008). These agents can be further divided into primary bacterial and viral pathogens, and secondary or opportunistic pathogens (Brockmeier *et al*., 2002; Maes *et al*., 2008). Adverse environmental and management conditions could exacerbate the severity of swine respiratory disease (Maes *et al*., 2008). Therefore, it would be very helpful to control swine respiratory diseases by clarifying the microbial communities in swine lungs and elucidating their relationship with lung lesions or their roles in promoting healthy lungs.

Still there are few studies on the lung microbes in pigs. Recently, a total DNA shotgun metagenomic analysis was performed using two lavage pools (ten samples each pool) from the lungs of pigs with suggestive signals of enzootic pneumonia and without any infection signals (Siqueira *et al*., 2017). This pilot work provided a microbiome overview in swine lungs under a field condition. They found that the most common families in the pneumonic swine's lungs were *Mycoplasmataceae*,* Flavobacteriaceae* and *Pasteurellaceae*, whereas in the carrier swine's lungs, the most common families were *Mycoplasmataceae*,* Bradyrhizobiaceae* and *Flavobacteriaceae*.

In our present study, we collected 20 pulmonary lavage‐fluid samples, extracted their DNAs, sequenced the V3‐V4 region of 16S rRNA gene and investigated their microbial communities. In addition, we recorded a lung‐lesion phenotype for these animals and tried to elucidate the relationship between the lung microbes and the extent of lung lesions. A better understanding of lung microbiome and its influence on healthy and diseased lungs will be conducive to pig breeding and provides important information for the diagnosis and treatment of pulmonary inflammation.

## Results

### Lung‐lesion phenotypic statistics and its effect on growth

**Figure 1:**
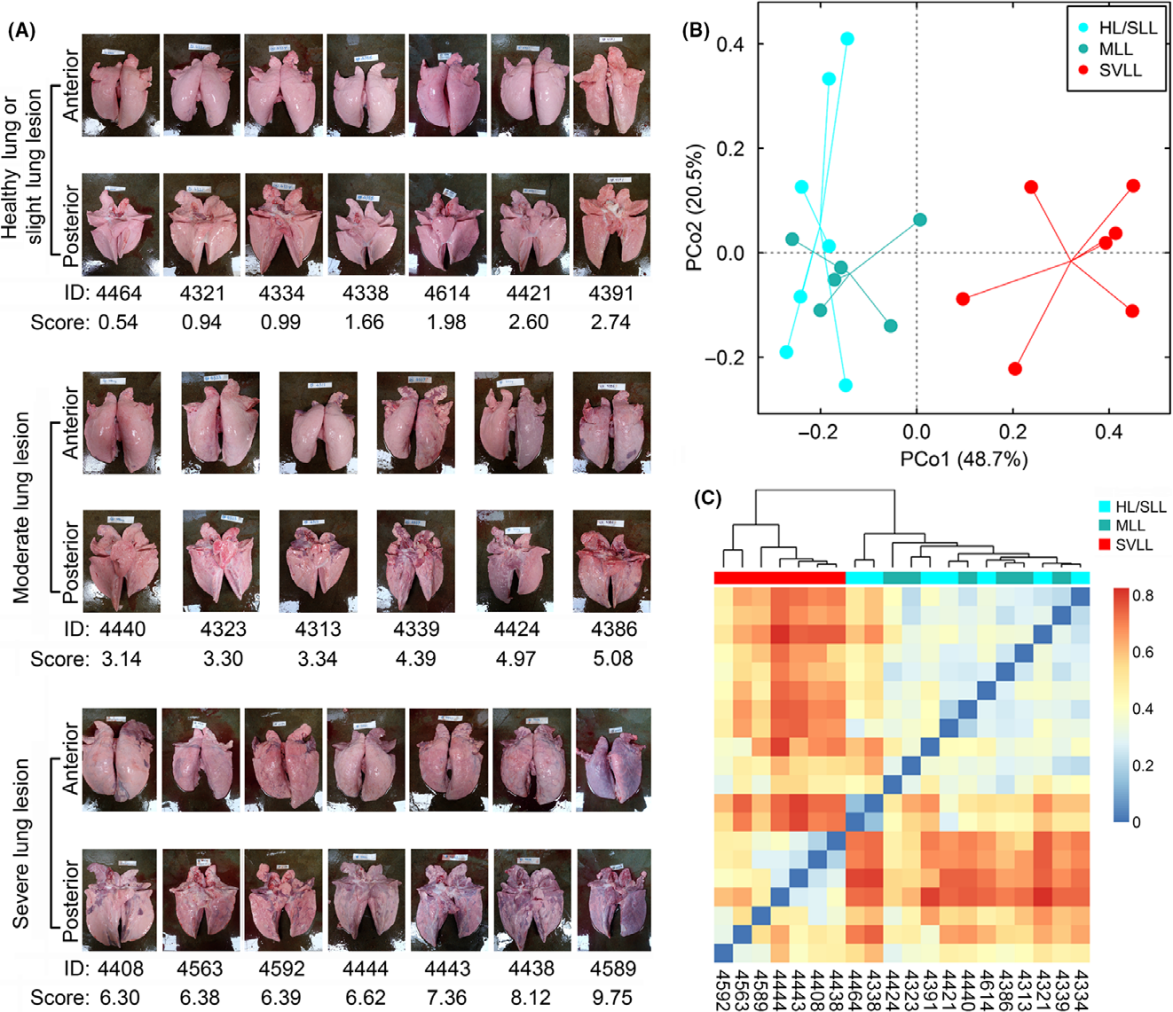
The collected swine lungs and their lung‐lesion scores and grouping. A. The anterior and posterior pictures of tested swine lungs and their lung‐lesion scores and grouping information. B. The principal coordinate analysis for lung microbiota in the tested samples based on the weighted UniFrac distances. C. Clustering and heatmap of the tested individuals based on the weighted UniFrac distances. HL/SLL represents the group of healthy lung or slight lung lesion; MLL represents the moderate lung‐lesion group; SVLL represents the severe lung‐lesion group.

According to the scoring criterion of lung lesion, we obtained the phenotypic values of lung lesions for the 20 tested pigs (Fig. 1A). The phenotypic values varied from 0.54 to 9.75. Based on the extent of lung lesions, we classified them into three groups: healthy lung or slight lung‐lesion (HL/SLL) group (lung‐lesion score < 3), moderate lung‐lesion (MLL) group (lung‐lesion score ≥ 3 and < 6) and severe lung‐lesion (SVLL) group (lung‐lesion score ≥ 6). The average lung‐lesion score of HL/SLL, MLL and SVLL groups was 1.71 ± 0.97, 3.83 ± 0.81 and 7.38 ± 1.36 respectively (Table 1). Meanwhile, we recorded body weights at the ages of birth, 120 and 240 days; average daily gains (ADG) were calculated from birth to 120 days and from 120 to 240 days (Table 1). A linear mixed‐effect model was used to estimate the association between lung lesions and growth traits. We found that lung lesions significantly affected the body weight at the age of 240 days (*P* = 0.044), and its negative effect on the ADG from 120 to 240 days was nearly significant (*P *=* *0.067, Table 1). There was no significant association between lung lesions and other growth traits, including birthweight, body weight at the age of 120 days and ADG from birth to 120 days.

**Table 1:** Descriptive statistics of lung‐lesion score, body weight and average daily gain^a^

Group	Score	Birthweight (kg)	Body weight (kg, D120)	Body weight (kg, D240)	ADG (kg per day, 0–120)	ADG (kg per day, 120–240)
HL/SLL	1.71 ± 0.97	1.32 ± 0.24	23.65 ± 2.61	90.78 ± 11.68	0.19 ± 0.02	0.56 ± 0.09
MLL	3.83 ± 0.81	1.06 ± 0.27	19.08 ± 4.39	79.03 ± 7.23	0.15 ± 0.03	0.50 ± 0.04
SVLL	7.38 ± 1.36	1.27 ± 0.26	22.30 ± 1.85	76.35 ± 14.77	0.18 ± 0.02	0.45 ± 0.12
*P*‐value^b^	/	0.650	0.228	0.044	0.212	0.067

a ADG, average daily gain; D120, the age of 120 days; D240, the age of 240 days; kg, kilogram; kg/day, kilogram per day.

b *P‐*value denotes the significance of association between birthweight, body weights or average daily gains and lung‐lesion score using a linear mixed‐effect model (See Materials and Methods).

### Biomass in swine lung

For each tested pig, almost the same volume of lung‐lavage fluid was collected ranging from 45 to 50 ml. We found the amount of DNA in lung‐lavage fluid was significantly highest in the SVLL group, which was three and nearly six times larger than that in the MLL group and the HL/SLL group respectively (Table 2). We used 2 μl of undiluted template DNA to perform PCR for 16S rRNA V3‐V4 region under same conditions and used 3 μl of PCR products (20 μl in total) to run electrophoresis; similarly, significantly higher PCR products were observed in the SVLL group than those in the HL/SLL and MLL groups (Table 2 and Fig. S1A).

**Table 2:** Summary of DNA mass in bronchoalveolar lavage fluid, PCR product of 16S rRNA V3‐V4 region, sequencing data and lung microbial structure identified in swine lungs

Group	DNA mass^a^ (ng)	PCR product (IntDen^b^)	Tag sequence number	OTU number	Genus number
HL/SLL	3735.7 ± 2889.7^a^	107.1 ± 107.1^a^	32682 ± 3508	734 ± 117^a^	155 ± 18^a^
MLL	7221.7 ± 4316.1^ab^	186.5 ± 183.4^a^	36130 ± 4943	777 ± 109^a^	164 ± 10^a^
SVLL	21504.3 ± 17784.5^b^	1296.9 ± 281.7^b^	36826 ± 3806	370 ± 119^b^	104 ± 21^b^
*P*‐value^c^	0.018	4.82 × 10^−9^	0.160	7.71 × 10^−6^	1.10 × 10^−5^

a Different letters show statistically significant differences between the tested groups.

b IntDen represents integrated density of PCR band.

c *P* value is the significance of the ANOVA test among the HL/SLL, MLL and SVLL groups.

### Analysis of negative controls

Following the same extraction and PCR procedures used for the lung lavage‐fluid samples, three negative controls were extracted using the same kit by the same laboratory technician and at the same laboratory. To be noted, negative controls were not processed on the same day as compared to the lung lavage‐fluid samples. Therefore, kit and/or laboratory contamination cannot be ruled out completely.

We sequenced these negative controls. We found that: (i) very low DNA mass was in the negative controls, and PCR products almost could not be detected by electrophoresis (Fig. S1B). (ii) The number of tag sequences in all negative controls (2244–3081) was much lower than that in the lung lavage‐fluid samples (27 272–43 674, Table S1); at the phylum level, the total phyla abundances in negative controls were obviously lower than those in the lung lavage‐fluid samples (Fig. S2). (iii) 20 genera were shared across all three negative controls, of which 15 genera were detected previously as background contamination from respiratory microbiota studies (Marsh *et al*., 2018) and five genera (*Agrobacterium*,* Phascolarctobacterium*,* p‐75‐a5*,* Mycobacterium* and *Hyphomonas*) were newly detected in our present study (Table S2). (iv) There was a strong difference in microbial profiles between the lung lavage‐fluid samples and negative controls based on weighted UniFrac distances (Fig. S3). (v) A total of 243 OTUs was detected in the negative controls, of which 59 OTUs have an average relative abundance of more than 0.5%. Of these 59 OTUs, 27 were shared between negative controls and lung lavage‐fluid samples. When we excluded the shared 27 OTUs from the original analysis of the lung lavage‐fluid samples, the sample relationship in the different groups remained almost unchanged (Fig. S4).

We did not find any evidence for bias or skew arising from the negative control sequences. Therefore, we kept all 20 lung lavage‐fluid samples and all qualified OTUs from these 20 samples for the next analyses.

### Identification and filtering of OTUs in swine lung

Clean sequences were obtained by removing low‐quality reads, primer and barcode from raw sequencing data, and then, paired clean sequences were merged together. Tag sequences were generated from merged clean sequences after OTUs clustering and chimera removing. In total, we obtained 703 337 tag sequences (an average of 35 167 per sample) for the lavage‐fluid samples of swine lungs (Table S1). Based on these tag sequences, we detected a total of 1382 OTUs. These OTUs were classified into microbial taxa, and a total of 35 phyla and 240 genera were identified. The summary of sequencing data and lung microbial structure identified in swine lungs was also shown in Table 2.

After removing the OTUs with an average relative abundance of < 0.01% and the OTUs only detected in one individual, we obtained 872 qualified OTUs in these pigs, which occupied 98.9% of the total clean reads. In the next analyses of group comparison and association test between lung lesions and lung microbiome, we used these 872 qualified OTUs.

### Microbial diversity in swine lung

We performed a principal coordinate analysis using the weighted UniFrac distances for these 20 lung lavage‐fluid samples. We found that the individuals with severe lung lesions clustered together and separated from those with healthy lung or slight lung lesions and the ones with moderate lung lesions at the first principal coordinate. And the samples with moderate lung lesions gathered together in the bottom left region of the PCoA plot; the samples with healthy lung or slight lung lesions ranged larger and some of them overlapped with the samples with moderate lung lesions (Fig. 1B). We also employed the weighted UniFrac distances to cluster the samples and draw a heatmap for these samples; the result of distance clustering was consistent with the PCoA analysis (Fig. 1C). Both results suggested that the relationship of microbial communities from different groups was generally consistent with the grouping based on the extent of lung lesions.

**Figure 2:**
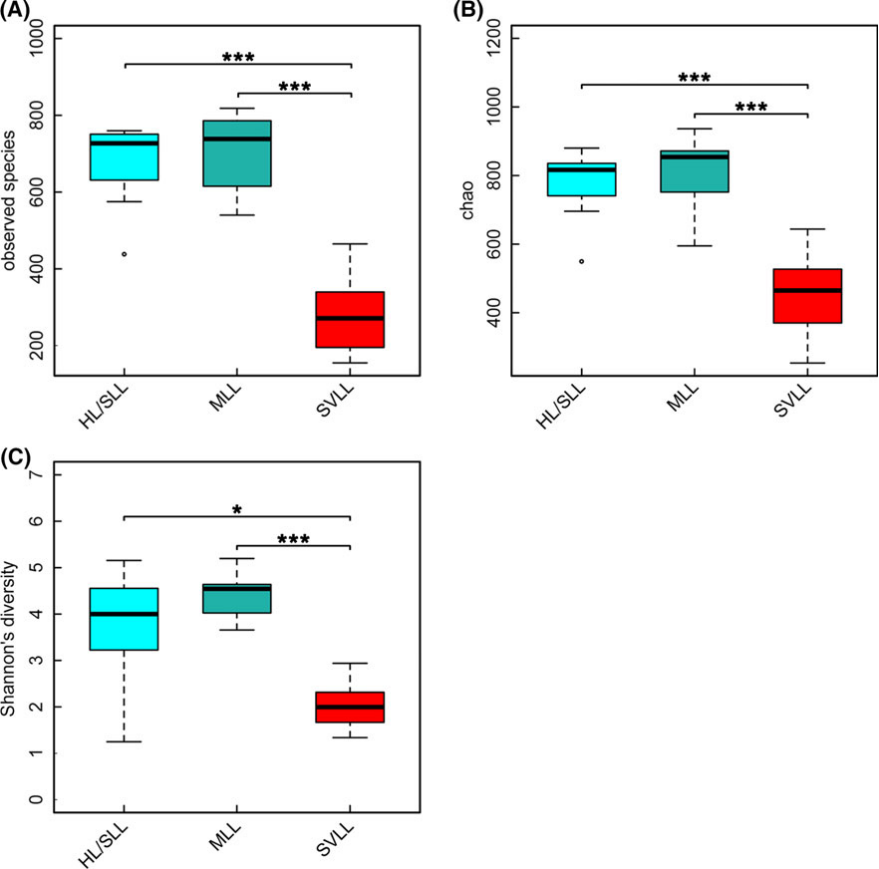
Alpha‐diversity of lung microbial communities for three different lung‐lesion groups.A. The lung microbial richness estimated by observed species.B. The lung microbial richness estimated by chao index.C. The microbial diversity evaluated by Shannon index. Significant differences were tested using Wilcoxon *t*‐test (*, 0.01 ≤ *P *<* *0.05; **, 0.001 ≤ *P *<* *0.01; ***, *P *<* *0.001).

We compared the alpha‐diversity of lung microbiota among these three groups with different lung lesions using the richness indices of observed species (Fig. 2A) and chao1 (Fig. 2B), and the diversity index of Shannon (Fig. 2C). We found that all three indexes showed significant difference between the severe lung‐lesion group and the group with healthy lung or slight lung lesions (*P *=* *4.39 × 10^−5^, 4.10 × 10^−4^ and 0.017 respectively) and between the severe lung‐lesion group and the moderate lung‐lesion group (*P *=* *2.46 × 10^−5^, 3.49 × 10^−4^ and 8.13 × 10^−6^ respectively), but no significant difference between the moderate lung‐lesion group and the group with healthy lung or slight lung lesions. The samples with severe lung lesions had a significantly lower alpha‐diversity.

### Composition of microbial communities in swine lungs

**Figure 3:**
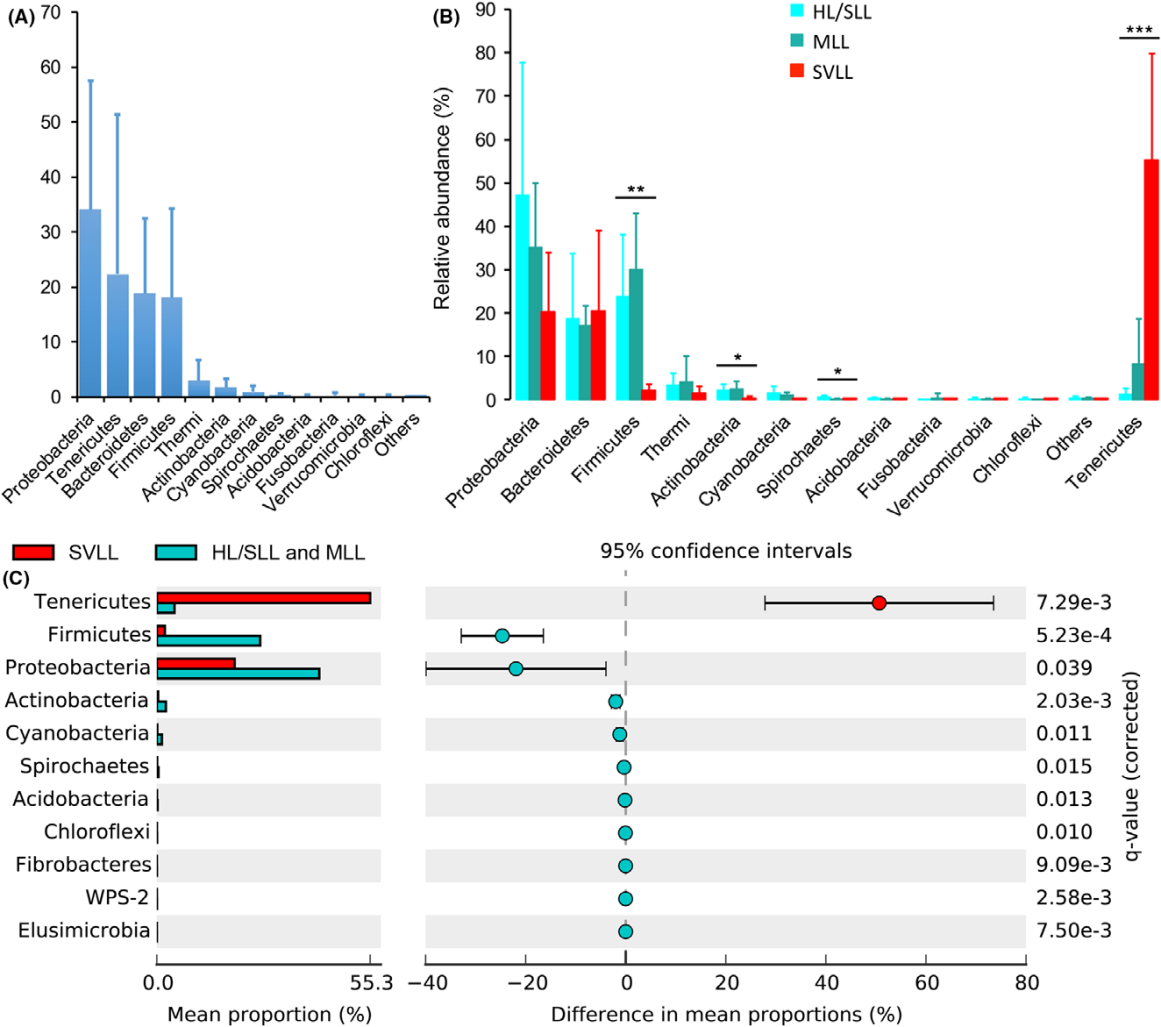
Overview and comparison of swine lung microbiota at the phylum level.A. The phyla with relative abundance more than 0.05% were shown. The phyla with relative abundance < 0.05% are summarized as ‘Others’.B. Comparison of twelve phyla with relative abundance more than 0.05% among the three groups with different lung lesions. HL/SLL represents the group of healthy lung or slight lung lesion; MLL represents the moderate lung‐lesion group; SVLL represents the severe lung‐lesion group. Significant differences were tested by the ANOVA analysis with Benjamini–Hochberg FDR correction (*, 0.01 ≤ *P *<* *0.05; **, 0.001 ≤ *P *<* *0.01; ***, *P *<* *0.001).C. Eleven significantly different phyla between the SVLL group and the other healthier‐lung groups, including four phyla with lower relative abundance < 0.05%. Welch's *t*‐test implemented in STAMP software was used; *q*‐values were corrected with Benjamini–Hochberg FDR method.

Using all 1382 OTUs, we identified a total of 240 genera representing 35 phyla in our tested swine lungs. There were twelve phyla with an average relative abundance of more than 0.05%, including *Proteobacteria*,* Tenericutes*,* Bacteroidetes*,* Firmicutes*,* Thermi*,* Actinobacteria*,* Cyanobacteria*,* Spirochaetes*,* Acidobacteria*,* Fusobacteria*,* Verrucomicrobia* and *Chloroflexi*. The total relative abundance of other 23 phyla was < 0.22%. Of these twelve high‐relative‐abundance phyla, *Proteobacteria* (34.2%), *Tenericutes* (22.3%), *Bacteroidetes* (18.8%) and *Firmicutes* (18.1%) were the most predominant in swine lungs (Fig. 3A), and four phyla of *Tenericutes* (*P *=* *1.46 × 10^−4^, ANOVA test with Benjamini–Hochberg FDR correction), *Firmicutes* (*P *=* *3.93 × 10^−3^), *Actinobacteria* (*P *=* *3.60 × 10^−2^) and *Spirochaetes* (*P *=* *3.45 × 10^−2^) showed significant differences among the HL/SLL, MLL and SVLL groups (Fig. 3B). Three phyla of *Proteobacteria* (47.3%), *Firmicutes* (23.8%) and *Bacteroidetes* (18.7%) were the most abundant in the HL/SLL group, which were also predominant in the MLL group (Fig. 3B). We made a comparison between HL/SLL and MLL groups and found that almost no significant difference was detected in the phyla level except for *Spirochaetes* (*P *=* *0.041). Nearly significant difference was observed for *Tenericutes* (*P *=* *0.078). The average relative abundance of *Spirochaetes* in the MLL group was lower than that in the HL/SLL group; the amount of *Tenericutes* had an increasing trend in the MLL group (Fig. S5). Notably, the composition of microbial communities in the SVLL group changed a lot compared to the HL/SLL and MLL groups. The relative abundance of *Tenericutes* was increased largely from a low proportion (1.3% in the HL/SLL group; 8.4% in the MLL group) to a higher level (55.2% in the SVLL group), while six phyla (*Proteobacteria, Firmicutes, Actinobacteria, Cyanobacteria, Spirochaetes* and *Acidobacteria*) were reduced significantly in the SVLL group (Fig. 3C). In addition, four phyla with an average relative abundance < 0.05% (*WPS‐2*,* Elusimicrobia*,* Fibrobacteres* and *Chloroflexi*) were also significantly reduced in the SVLL group (Fig. 3C).

**Figure 4:**
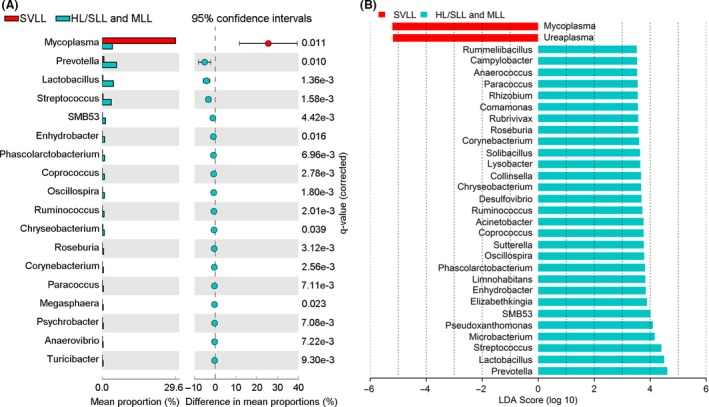
Comparison of lung microbiota between the severe lung‐lesion group and the healthier‐lung groups at the genera level.A. Eighteen genera with significant difference between the SVLL group and the HL/SLL and MLL groups. The relative abundances of these eighteen genera were more than 0.5%; the rest significantly different genera with the relative abundance < 0.5% were listed in Fig. S6. Significant difference (*P *<* *0.05) between the SVLL group and the healthier‐lung groups was tested by Welch's *t*‐test with Benjamini–Hochberg FDR correction implemented in the STAMP software.B. Thity‐one significantly different genera with LDA threshold > 3.5. The linear discriminant analysis (LDA) scores were estimated between the SVLL group and the HL/SLL and MLL groups in the LEfSe analysis.

We further examined the microbial communities at the genus level in these pigs. After filtering out the genera with an average relative abundance < 0.01%, we obtained 137 genera for further analysis (Table S3). Among them, *Mycoplasma* (13.0%), *Methylotenera* (10.9%), *Ureaplasma* (9.2%), *Phyllobacterium* (5.3%), *Prevotella* (4.0%), *Sphingobium* (3.2%), *Lactobacillus* (3.0%), *Thermus* (2.7%), *Streptococcus* (2.4%) and *Haemophilus* (1.8%) were the top ten genera. Of these 10 genera, significant differences in the relative abundance were observed in *Mycoplasma*,* Lactobacillus* and *Streptococcus* among the HL/SLL, MLL and SVLL groups. Thirteen other genera with low relative abundance also showed significant differences among these three groups (Table S3). The most predominant genera in each group of HL/SLL, MLL and SVLL were shown in Table S3. Of these predominant genera, no significant difference was detected between the HL/SLL and MLL groups. When we compared the microbial communities in the SVLL group with those in the HL/SLL and MLL groups, 73 genera with significant differences were observed (Fig. 4A and Fig. S6), which was highly consistent with the result at the phyla level. Most of these genera were reduced in the SVLL group including the predominant genera of *Prevotella*,* Lactobacillus* and *Streptococcus*, while only one genus of *Mycoplasma* was significantly increased in the SVLL group. It was notable that five animals in the SVLL group contained high abundance of *Ureaplasma* and three animals in the SVLL or MLL group contained high abundance of *Haemophilus* (Fig. S7), which suggested that the five pigs might accompany with the infection of *Ureaplasma* and the three pigs might suffer from a secondary infection of *Haemophilus* sporadically.

We also performed a LEfSe analysis to identify the differential bacterial genera between the SVLL group and the HL/SLL and MLL groups. Ninety‐seven significantly differential genera (*P *<* *0.05) were detected. The bacterial genera with LDA score more than 3.5 are shown in Fig. 4B. Two genera of *Mycoplasma* and *Ureaplasma* were enriched in the SVLL group (LDA score = −5.21 and −5.09 respectively). Instead, there were 29 genera showing higher abundances in the healthier‐lung group, including *Prevotella*,* Lactobacillus*,* Streptococcus*,* Microbacterium* and *Pseudoxanthomonas* (Fig. 4B). These results of differential genera between the SVLL group and the HL/SLL and MLL groups were mostly consistent with those detected by the STAMP analyses.

### Microbial taxa associated with lung lesions

**Figure 5:**
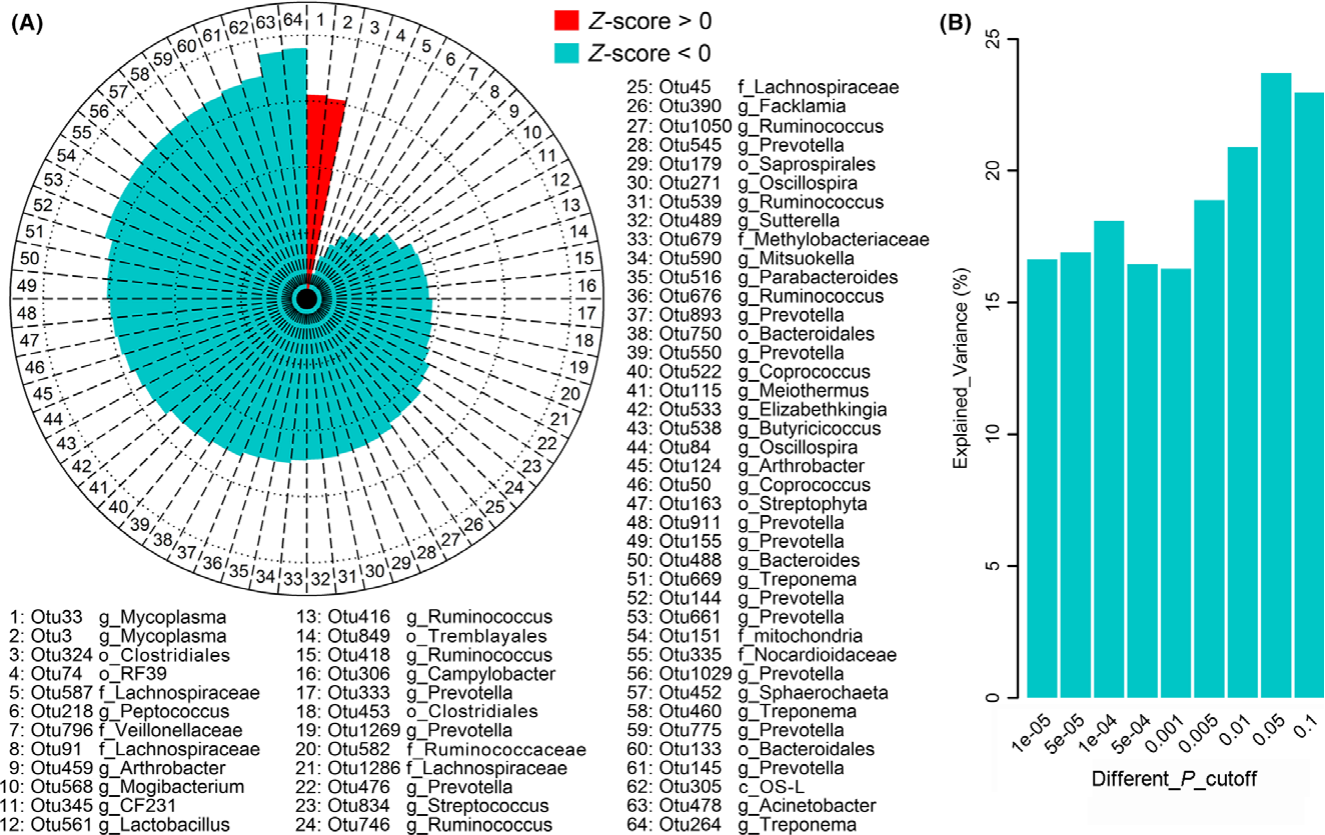
Correlation between OTUs and lung lesions and lung microbiome's contribution to lung lesions. A. Sixty‐four lung‐lesion‐associated OTUs (FDR < 0.1). The red leaf indicates positive correlation between OTUs and lung lesions, and the cyan leaf denotes negative correlation. OS‐L: Armatimonadetes member, OS Type L. B. The contribution of lung microbiome to lung lesions. The bar shows the variation of pulmonary lesions explained by lung microbes at different significance levels from 1 × 10^−5^ to 0.1.

In the present study, 64 OTUs were identified and significantly associated with lung‐lesion score using two‐part model association analysis (FDR < 0.1). Of these 64 OTUs, two were positively associated with the extent of lung lesions and 62 had negative correlations with lung‐lesion score (Fig. 5A). In details, 42 associated OTUs (65.6%) were identified by the binary analysis (presence/absence), five associated OTUs (7.8%) were detected by quantitative analysis (the abundance of bacteria), and the other 17 associated OTUs (26.6%) were identified by meta‐analysis (both presence/absence and the abundance of bacteria).

These 64 lung‐lesion‐associated OTUs were classified to microbial taxa as shown in Fig. 5A. In summary, one OTU was classified to the class level, eight OTUs to the order level, nine to the family level, forty‐two to the genus level, only four to the species level.

Notably, both OTUs positively associated with the extent of lung lesions (*P *=* *1.94 × 10^−3^ and 2.19 × 10^−3^) were classified to *Mycoplasma*; thirteen OTUs classified to *Prevotella* and six OTUs classified to *Ruminococcus* showed negative associations with lung‐lesion score.

### Contribution of microbiota to lung lesions

To investigate how much degree of lung‐lesion score was contributed to by the lung microbiome, we conducted a 1000 randomizations cross‐validation analysis by splitting the data set randomly into a 70% discovery set and a 30% validation set at the OTU level. We found that OTUs identified at the 1 × 10^−5^ level in the discovery set could explain 16.6% phenotypic variation of lung‐lesion extent. When the significance threshold of association increased to *P *=* *0.05 and the risk model included more OTUs, the explained variance increased to 23.7% (Fig. 5B).

### Association between predicted microbial function capacity and lung lesions

We performed MaAsLin analysis to investigate the correlation between predicted function capacity of lung microbiome and lung lesions and identified 22 KEGG pathways related to lung lesions. Of these 22 pathways, 15 pathways had negative correlation with lung lesions, while seven pathways showed positive correlation with lung lesions (for details see Table S4).

**Figure 6:**
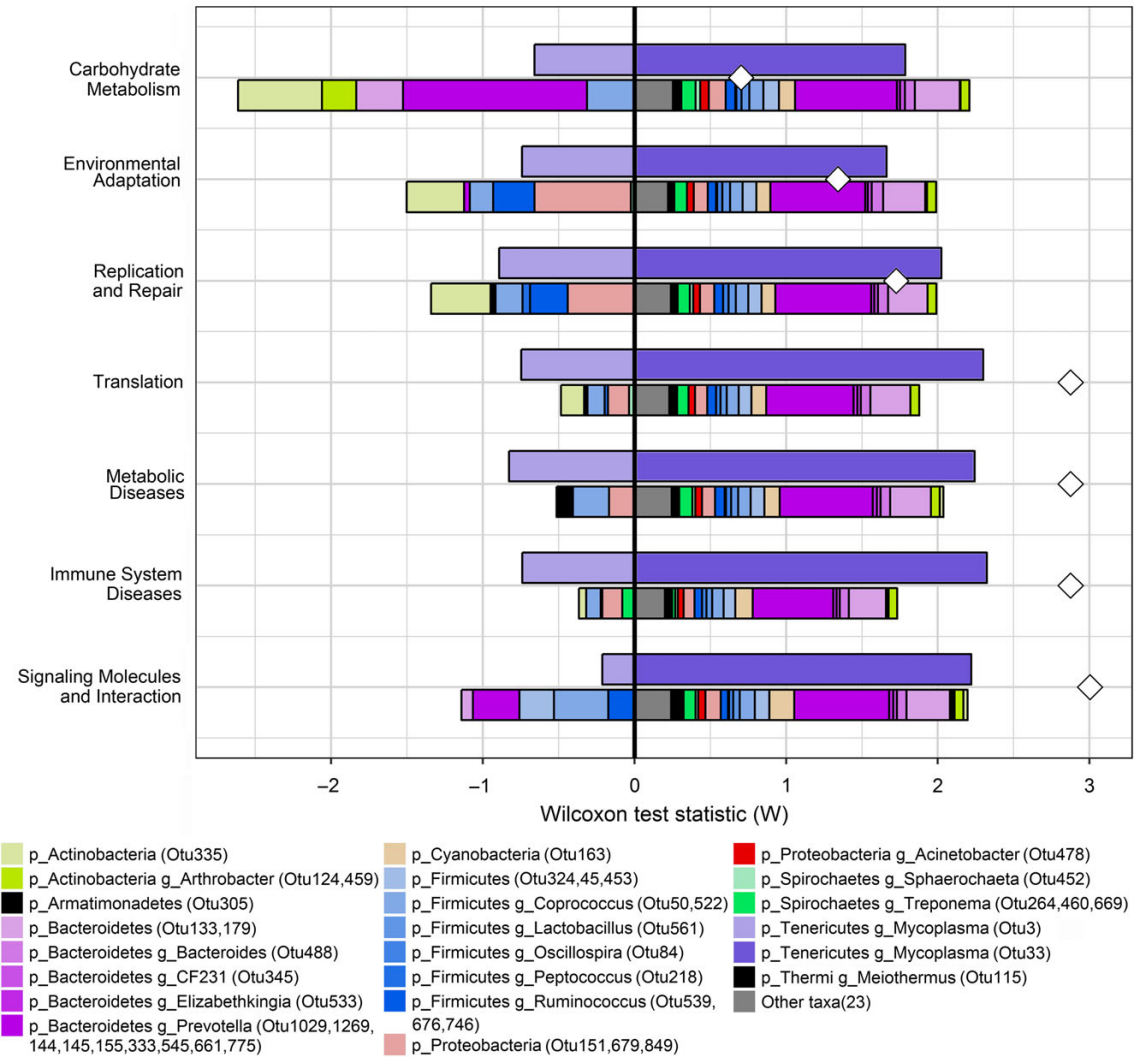
The contribution of lung‐lesion‐associated OTUs to the functional shifts of lung‐lesion‐associated KEGG pathways. For each pathway, the sum of OTU contribution scores matches the observed OTU‐based functional shift score; for each pathway, the bars on the upper‐right and left, lower‐right and left parts centred around the *Y* axis represent severe lung‐lesion (SVLL)‐associated OTUs driving and attenuating functional shifts, healthy lung or slight lung‐lesion (HL/SLL)‐associated OTUs driving and attenuating functional shifts, respectively. The white diamond indicates the taxa‐based functional shift score.

Further, we estimated the contribution of the lung lesion‐associated OTUs to functional shifts of lung microbial community. As shown in Fig. 6, OTU1029, 1269, 144, 145, 155, 333, 545, 661 and 775 (g_*Prevotella*), OTU533 (g_*Elizabethkingia*), OTU345 (g_*CF231*), OTU488 (g_*Bacteroides*) and OTU133, 179 (p_*Bacteroidetes*) mostly contributed to the function capacities associated with healthy lung, especially to the diseases‐ and metabolism‐associated function terms. Notably, most of the other taxa (*n* = 23) with low relative abundance < 0.01% contributed to driving functional shifts in the health lungs, which suggested that these microbes with low relative abundance might play important roles in keeping lung healthy. On the contrary, OTU33 (g_*Mycoplasma*) was detected as a taxonomic driver of functional shifts in the lungs with severe lung lesions.

## Discussion

Previously, the healthy lungs were considered sterile on the basis of classical culture studies (Baughman *et al*., 1987; Thorpe *et al*., 1987). With the novel culture‐independent techniques applying to microbial identification and characterization (Hilty *et al*., 2010), it was clear that the healthy lung harbours a high diversity of bacteria. In the last few years, the research on the lung microbiome was increasing rapidly, including characterization of the normal lung microbiome (Charlson *et al*., 2011; Erb‐Downward *et al*., 2011), investigation of the microbial communities at different developmental stages (Kostric *et al*., 2018) and exploration of the lung microbiome in disease states (Hilty *et al*., 2010; Erb‐Downward *et al*., 2011; Guss *et al*., 2011; Huang *et al*., 2011; Scher *et al*., 2016).

The lung microbiota is a complex variety of microbes found in the lower respiratory tract, and some lung microbes may play important roles in keeping lung or body health (Villena *et al*., 2005; Khailova *et al*., 2014). More recently, a preliminary overview of swine lung microbiome was provided by investigation of two lung lavage pools using a total DNA shotgun metagenomic analysis (Siqueira *et al*., 2017). Niederwerder (2017) made a review about the microbiome role in swine respiratory disease. The review majorly discussed associations of the microbiome with growth and immunity during respiratory disease infection and the microbiome role in porcine reproductive and respiratory syndrome; it also suggested that modulation of the microbiome might be an alternative tool in the control of swine respiratory disease.

In the present study, we first investigated the microbial communities in swine lungs. *Proteobacteria* (47.3%), *Firmicutes* (23.8%) and *Bacteroidetes* (18.7%) were the most dominant lung phyla in swine healthy lungs, which shows a similar phylum distribution as previously reported for lung tissue microbiota in human (Erb‐Downward *et al*., 2011; Beck *et al*., 2012) and in mice (Barfod *et al*., 2013; Yun *et al*., 2014). There was a large different phylum distribution between healthy and severe‐lesion lungs, which suggested that the community membership of the lung microbiome was changed in the disease state. Same in human, the microbial ecology alters greatly in lung from health to chronic diseases such as chronic obstructive pulmonary disease, cystic fibrosis and idiopathic pulmonary fibrosis (Dickson *et al*., 2014).

We found that lung lesions showed negative effect on the body weights at the age of 240 days and average daily gains from 120 to 240 days in these pigs. This result was generally consistent with previous reports that swine enzootic pneumonia, a chronic and endemic respiratory disease caused by *Mycoplasma hyopneumoniae*, could lead to decreased growth rate and reduced feed efficiency in pigs (Thacker *et al*., 2002). It infers that lung lesions of these test pigs might be possibly related to swine enzootic pneumonia as these pigs were immunized with several common virus vaccines, such as classical swine fever, porcine circovirus type II disease, pseudorabies, foot‐and‐mouth disease and porcine reproductive and respiratory syndrome. We also found that there was more biomass in the pulmonary lavage fluid of severe‐lesion lungs than healthy lungs or slight‐lesion lungs, but the microbial diversity in the severe‐lesion lungs was lower than that in the healthy lungs or slight‐lesion lungs. Reduced microbial diversity along with severity of airway obstruction was also observed in some human respiratory diseases, like cystic fibrosis (CF) disease (Cox *et al*., 2010; Zhao *et al*., 2012), chronic obstructive pulmonary disease (COPD; Erb‐Downward *et al*., 2011).

We identified 64 OTUs significantly associated with lung lesions using two‐part model association analysis. Of these OTUs, two OTUs positively associated with lung lesions, which were classified to *Mycoplasma* and might contribute to the development of lung lesions; the other OTUs (*n* = 62) negatively associated with lung lesions, which inferred that these bacteria might play roles in keeping lungs healthy or protecting lungs against pathogenic microorganisms. To our knowledge, it is the first time that an association analysis between lung lesions and lung microbes in mammal was performed.

Two genera of *Mycoplasma* and *Ureaplasma* were enriched in the severe‐lesion lungs, and two OTUs classified to *Mycoplasma* showed positive association with lung lesions. Currently, many studies about *Mycoplasma*, especially about *Mycoplasma hyopneumoniae*, have been reported, including its isolation (Goodwin *et al*., 1965; Mare and Switzer, 1965; Whittlestone, 1973), taxonomical characterization (Tajima and Yagihashi, 1982; Blanchard *et al*., 1992; Dybvig and Voelker, 1996; Razin, 2006), genomic sequencing (Minion *et al*., 2004; Vasconcelos *et al*., 2005; Liu *et al*., 2011), colonization of swine respiratory tract (Blanchard *et al*., 1992; DeBey and Ross, 1994; Simionatto *et al*., 2013) and prevalence in pigs (Sibila *et al*., 2007; Grosse Beilage *et al*., 2009). We further annotated the two OTUs by aligning their sequences to the NCBI nt database. OTU3 was highly similar to *Mycoplasma hyopneaumoniae* (e.g. *Mycoplasma hyopneaumoniae* Strain 232, GenBank Access No. AE017332.1) with 100% query cover and 99% identity, and OTU33 is likely to be *Mycoplasma flocculare* (e.g. *Mycoplasma flocculare* ATCC 27399, GenBank Access No. CP007585.1) also with 100% query cover and 99% identity. There are many reports on *Mycoplasma hyopneaumoniae* but few studies on *Mycoplasma flocculare*. *Mycoplasma flocculare* was first identified in 1972 and was generally considered to be a commensal colonizer of the porcine nasopharynx but an opportunist pulmonary pathogen in coinfections with *Mycoplasma hyopneumoniae* (Meyling and Friis, 1972). Recently, complete genome sequence of *Mycoplasma flocculare* strain Ms42^T^ was reported, which would enable more detailed analyses of genome structure and plasticity among the *Mycoplasma* species (Calcutt *et al*., 2015). In this study, both *Mycoplasma hyopneumoniae* and *Mycoplasma flocculare* showed strong association with lung lesions. Interestingly, the taxonomic driver of functional shifts in the severe lung‐lesion lungs was evaluated to be *Mycoplasma flocculare*, not *Mycoplasma hyopneumoniae*, which suggested the pathogenesis of *Mycoplasma flocculare* was also valuable to be further studied. The genus *Ureaplasma* belongs to the family *Mycoplasmataceae* in the order *Mycoplasmatales*, which was often detected in the genitourinary samples associated with urogenital diseases (Yoshida *et al*., 2003). *Ureaplasma* infection can occur in extragenital sites and lower respiratory tract (Waites *et al*., 2005). *Ureaplasma* infection of the lower respiratory tract could increase twice the risk of the development of chronic lung disease or death in the very low‐birthweight infants (Cassell *et al*., 1988).

Sixty‐two OTUs were detected to be negatively associated with lung lesions. Most OTUs (*n* = 49) were with low relative abundance < 0.05%, which suggested that the healthy lungs were colonized with many divergent beneficial bacteria with small abundances. Of these 62 OTUs, thirteen were annotated to *Prevotella*, six to *Ruminococcus*. These two genera were often detected in the gut microbiome and helped the breakdown of protein and carbohydrate foods (Ramakrishna, 2013), their functions in lung need further studies. Among these 62 OTUs, some microbes were previously reported to be beneficial, like *Lactobacillus*, as it protects the host against potential invasions by pathogens (Servin, 2004). In mice, it was reported that *Lactobacillus* isolates from the murine lung lead to alterations in alveolar numbers and size and mucus production (Yun *et al*., 2014). However, some microbes were reported to be pathogenic bacteria, such as *Elizabethkingia* (Jean *et al*., 2014), *Treponema* (Salazar *et al*., 2002) and *Streptococcus* (Wertheim *et al*., 2009). Possibly all these negative‐associated microbiota in the healthy lungs could develop and maintain a micro‐ecological balance in the lung environment.

In our present study, cross‐validation analysis showed that lung microbiota explained 23.7% phenotypic variance of lung lesions. This finding suggested the lung microbiota had a large effect on promoting lung health or resulting in lung lesions.

To be noted, there were two major limitations in our present study. First, the number of samples we collected here was small. In future, the sample size should be increased to get more robust results, especially for validation of the low abundant microbiota in swine lungs. Second, negative controls were not processed on the same day as compared to the lung lavage‐fluid samples. Therefore, kit and/or laboratory contamination cannot be ruled out completely.

## Materials and methods

### Experimental animals

A total of 20 lung lavage‐fluid samples were collected from nine males and 11 females, which were the six‐generation offspring of a population crossed by eight pig breeds Erhualian, Laiwu, Bamaxiang, Tibetan, White Duroc, Landrace, Large White and Pietrain. The 20 pigs were randomly selected from five batches of 211 slaughtered pigs, which were raised at a farm in Nanchang (28°50′34″N, 115°48′46″E), China, under natural conditions. These pigs were immunized with seven common vaccines, including those for classical swine fever and porcine circovirus type II disease at 15 days, streptococcal disease and pseudorabies at 24 days, foot‐and‐mouth disease at 32 days, porcine reproductive and respiratory syndrome and haemophilus suis at 40 days.

All pigs were under the same feeding procedures and management conditions. At the age of around 45 days, these pigs were transferred to fattening houses in 20 head‐to‐head pens (4 × 7 m), where each pen housed 10–15 pigs. These pigs were provided *ad libitum* water and corn–soybean feed. The corn–soybean feed contains 3100 kJ digestive energy, 14–17% crude protein, 0.85% lysine, 0.4–1.2% calcium, 0.35% phosphorus and 0.3–0.8% salt. All pigs did not receive any antibiotic treatment at least 2 months before slaughter.

### Sample collection

After fasting overnight, pigs were slaughtered at the age of 240 ± 3 days. All viscera of pigs were taken out immediately after slaughter, then the tracheas were cut‐off, and the whole lungs were separated out. Sterile PBS buffer (137 mM sodium chloride, 2.7 mM potassium chloride, 10 mM phosphate and 2 mM potassium phosphate) was poured into lungs, which were repeatedly and gently kneaded for 2 min. The lavage fluid from lungs was collected into 50‐ml sterile centrifugation tubes, and kept in ice, then taken back to our laboratory from the slaughterhouse within 1 h. For each sample, 45–50 ml of lavage fluid was collected, and the pellet of lung‐lavage fluid was transferred into a 2 ml sterile centrifugation tube using 4000 *g* centrifugation for 30 min at 4°C. Then, the pellet was used for immediate DNA extraction and or stored at −80°C until DNA extraction. Three sterile PBS buffer samples served as the negative controls.

### Phenotypic measurement and association

Each lung was laid at a bright place and was taken photographs for both anterior and posterior sides using a digital camera. The extent of lung lesions for each pig was estimated according to its lung photo with the following scoring criterion: (i) The lung was divided into seven parts, including left and right apical lobe, left and right cardiac lobe, left and right diaphragmatic lobe and intermediate lobe. For each lung part, the scores were assigned as zero, one, two, three, four and five, corresponding to the lung‐lesion proportions of < 5%, 5–15%, 15–25%, 25–35%, 35–45% and more than 45% respectively (Zou *et al*., 2013). (ii) Based on the volume size of each part, the weights were assigned as 5%, 6%, 7%, 9%, 32%, 36% and 5% for left and right apical lobe, left and right cardiac lobe, left and right diaphragmatic lobe and intermediate lobe respectively (Zou *et al*., 2013). Finally, the scores of 13 parts in both anterior and posterior sides multiplying their weights were summed as the overall score of lung lesions for each individual.

In addition, we recorded the birthweight, body weights at the ages of 120 and 240 days for each pig; we also obtained the daily gain from birth to 120 days and from 120 to 240 days for each pig.

A linear mixed‐effect model was used to examine the association between lung‐lesion score and birthweight, body weight, average daily gain. The model follows as: *y *= *score* + *batch* + *pen* + *e*, where *y* is birthweight, body weight at the age of 120 or 240 days, or average daily gain from birth to 120 days or from 120 to 240 days, *score* is lung‐lesion score, *batch* and *pen* are the environmental factors, *e* is the random residual.

### DNA extraction, PCR and sequencing

The DNA of bronchoalveolar lavage‐fluid pellet was extracted using QIAamp DNA Stool Mini Kit (Qiagen, Hilden, Germany) according to the manufacturer's instructions. The V3‐V4 region of 16S rRNA gene was amplified with barcode‐indexed universal bacterial primers (forward primer 338F, 5′‐ACTCCTACGGGAGGCAGCAG‐3′ and reverse primer 806R, 5′‐GGACTACHVGGGTWTCTAAT‐3′; Fadrosh *et al*., 2014). PCR was carried out in triplicate using the 20 μl reactions containing 10 ng of template DNA, 4 μl of fivefold FastPfu Buffer, 2 μl 2.5 mM dNTPs, 0.4 μl FastPfu Polymerase, 0.8 μl 5 μM each primer, 0.2 μl BSA and 9.8 μl ddH_2_O. The PCR was performed by one cycle with pre‐denaturation at 95°C for 3 m, 28 cycles with denaturation at 95°C for 30 s, annealing at 55°C for 30 s and elongation at 72°C for 45 s, last cycle with elongation at 72°C for 10 m, and keeping at 10°C until halted by user. A negative control in the form of a PCR‐amplified ultrapure water sample was included for each plate. For quantitation of PCR products, electrophoretic gels were scanned and the integrated density of each PCR band was determined using the ImageJ program (NIH Image, Bethesda, MA, USA).

Amplicons were extracted from 2% agarose gels and purified using the AxyPrep DNA Gel Extraction Kit (Axygen Biosciences, Union City, CA, USA) according to the manufacturer's instructions; the quantity was determined by a QuantiFluor‐ST handheld fluorometer (Promega, Madison, WI, USA). Purified amplicons were pooled in equimolar amounts and paired‐end sequenced on an Illumina MiSeq sequencing platform (Illumina, San Diego, CA, USA) according to the standard protocols (Caporaso *et al*., 2012) by Majorbio Bio‐Pharm Technology Co. Ltd. (Shanghai, China). Three negative controls were extracted same as lung lavage‐fluid samples following the same PCR procedures; then, purified amplicons of negative controls were paired‐end sequenced same as lung lavage‐fluid samples.

### Bioinformatics analysis of sequencing data

The raw fastq files were demultiplexed, quality‐filtered by Trimmomatic (Bolger *et al*., 2014) and merged by FLASH v1.2.11 (Magoc and Salzberg, 2011) with the following criteria: (i) the reads were truncated at any site receiving an average quality score < 20 over a 50 bp sliding window. (ii) Primers were exactly matched allowing 2‐nucleotide mismatching, and reads containing ambiguous bases were removed. (iii) Sequences whose overlap longer than 10 bp were merged according to their overlap sequence. Operational taxonomic units (OTUs) were clustered with a cut‐off of 97% similarity using UCLUST cluster algorithm implemented in USEARCH v7.0.1090 (Edgar, 2010), and chimeric sequences were identified and removed using UCHIME. The taxonomy of each 16S rRNA gene sequence was analysed by RDP v2.2 classifier program (Wang *et al*., 2007) against a primer‐specific version of the GreenGenes v13.5 reference database (DeSantis *et al*., 2006). Using TaxMan (Brandt *et al*., 2012), the primer‐specific reference database containing only reference entries that matched the selected primers was created as previously described (Fu *et al*., 2015).

### Microbial diversity analysis

The alpha‐diversity indexes of observed species, chao1 and Shannon index were calculated by Mothur v1.31.2 software (Schloss *et al*., 2009). Before this analysis, we rarefied sample tag sequence size to an uniform depth of 20 000 sequences using the rarefy function in the R package of Vegan v2.4‐2 (Dixon, 2003). Comparison of alpha‐diversity indexes among the groups with different lung lesions was performed by Wilcoxon t‐test. Beta diversity for each sample pair was measured using QIIME v1.8.0 (Caporaso *et al*., 2010) with weighted UniFrac distances. In the analysis of beta diversity, tag sequence sizes of all the samples were rarefied to 75% of the minimum tag sequence number among the lung lavage‐fluid samples (here, the minimum tag sequence number is 27 272, Table S1).

Comparison of relative abundances of lung bacterial phyla and genera between the severe‐lesion lungs (lung‐lesion score ≥ 6) and the other non‐severe‐lesion lungs (lung‐lesion score < 6) was performed by STAMP software (Parks *et al*., 2014). Before group comparison, the qualified OTUs were obtained by removing the OTUs with an average relative abundance of < 0.01% and only detected in one individual among the lung lavage‐fluid samples. Linear discriminate analysis effect size (LEfSe) (Segata *et al*., 2011) was also employed to identify the bacteria enriched in the severe‐lesion lungs and the other non‐severe‐lesion lungs.

### Two‐part model for association analysis

Considering the quantitative characterization of the lung‐lesion scores, an association study was also performed between the phenotype of lung lesion and the relative abundance of bacteria. Before association analysis, the qualified OTUs were obtained with the filtering criteria same as group comparison analyses. The residuals of lung‐lesion score corrected by the effects of gender, batch and pen were used for the association study. Due to the non‐normal distribution of the relative abundances of OTUs in the test pigs, association studies were performed with a two‐part model method as described previously (Fu *et al*., 2015). Briefly, the two‐part model association analysis included both binary and quantitative model. The binary model describes a binomial analysis that tests for association of detecting a microbe with lung‐lesion score. The quantitative model tests for association between the abundance of the detected microbes and lung‐lesion score. To combine the effect of both binary and quantitative features, a meta‐analysis was performed using an unweighted Z method. The final association *P* value was set as the minimum of *P* values of binary, quantitative and meta‐analysis. The distribution of the association *P* values could be skewed. To reduce distortion, 1000 permutation tests were performed to control the false discovery rate (FDR). For each permutation, we randomized the lung microbial composition across individuals and performed the 2‐part analysis on permuted data. At a certain *P* cut‐off, the average number of the detected significance (*N*_0_) in 1000× permutations was defined as the false positive, and its ratio to the detected positive (*N*_1_) in the real analysis was the FDR. We controlled the FDR at 0.1.

### Estimating the contribution of lung microbiome to lung lesions

The contribution of microbiome to lung lesion was similarly estimated as described previously (Fu *et al*., 2015; He *et al*., 2016). We split the data randomly into a 70% discovery set and a 30% validation set. In the discovery set, a total of *n* number of significantly associated OTUs was identified at a certain *P* value, and the effect sizes of binary and quantitative features of each OTU (*β*_1_ and *β*_2_) were estimated. Then, the risk of the lung microbiome on lung lesions (*r*_m_) for each animal in the validation set was calculated using an additive model:

$$r_{m}=\sum_{j=1}^{n}\left(\beta_{1}+b_{j}+\beta_{2j}q_{j}\right),$$

where *b* is a binary feature in the binary model and *q* is a quantitative feature in the quantitative model of two‐part model described above, *j* is the number of OTUs varied from 1 to *n*. The phenotypic variation of lung lesions explained by the lung microbiome was represented as Pearson correlation coefficient (*R* ^2^) between lung‐lesion score and *r*_m_, after correcting for sex. To ensure the stability of the estimation, we repeated the cross‐validation by 1000× and calculated the average value of the explained variation. Considering many microbes that may contribute a small effect but may not be confidently detected at FDR ≤ 0.1, we performed this analysis at a series of different significant *P* levels ranging from 1 × 10^−5^ to 0.1.

### Functional prediction of lung microbiome

Functional capacity of microbial community was predicted using PICRUSt online Galaxy version (Langille *et al*., 2013). The closed reference OTU table was generated from quality control reads in QIIME v1.8.0 against the Greengenes database v13.5 (DeSantis *et al*., 2006). OTU normalization, metagenome prediction and function categorization based on the secondary‐class Kyoto encyclopaedia of genes and genomes (KEGG) pathways were performed by PICRUSt according to a standard analysis process. Correlation between lung‐lesion scores and relative abundances of KEGG pathways was calculated by MaAsLin online Galaxy version (Morgan and Huttenhower, 2014). The significant threshold was set at FDR < 0.05.

### Identifying the taxonomic drivers of functional shifts

To evaluate the contribution of the lung lesion‐associated OTUs to functional shifts of lung microbiome, we used FishTaco analysis to establish the correlation between lung lesion‐associated OTUs and functional capacities as described by Manor and Borenstein (2017). Because FishTaco software has been only used to treat with the dataset from case–control study, we chose those samples in the healthier‐lung (lung‐lesion score < 3) group (control) and in the severe lung‐lesion (lung‐lesion score ≥ 6) group (case) for this analysis. The profiles of the relative abundances of lung lesion‐associated OTUs and predicted function capacities were inputted into FishTaco software. Multi_taxa module was run to assess the taxonomic contribution to functional shifts. The output result was visualized using ggplot2 in R package.

## Conflict of interest

None declared.

## Author contributions

TH and HA wrote the manuscript; TH analysed the data; TH, MZ, XT, JC and GY collected the samples and performed the experiments; BY and SX helped to collected the samples; SF and YG helped to analyse the data; HA and LH conceived and designed the experiments; HA, LH and CC revised the manuscript.

## Ethics statement

All animal works were conducted according to the guidelines for the care and use of experimental animals established by the Ministry of Agriculture of China. Animal Care and Use Committee in Jiangxi Agricultural University specially approved this project.

## Supporting information


**Fig. S1**. PCR results of 16S rRNA V3‐V4 region for 20 bronchoalveolar lavage fluid samples and 3 negative controls.

**Fig. S2.** Microbial communities at the phylum level in 20 bronchoalveolar lavage fluid samples and three negative controls.

**Fig. S3.** The principal component analysis for 20 bronchoalveolar lavage fluid samples and three negative controls based on the weighted UniFrac distances.

**Fig. S4.** The principal component analyses for original OTUs of lung microbiota versus the new set of OTUs removing 21 OTUs shared by negative controls.

**Fig. S5.** Comparison of *Spirochaetes* and *Tenericutes* between the HL/SLL group and the MLL group.

**Fig. S6.** Comparison of lung bacterial genera with low relative abundances of < 0.5%.

**Fig. S7.** The relative abundance of *Ureaplasma* and *Haemophilus*.

**Table S1.** Information of the raw data and the OTU data for all Lung lavage‐fluid samples and negative controls.

**Table S2.** The relative abundance of genera detected in three negative controls.

**Table S3.** The composition of lung microbiome for each group at the genus level.

**Table S4.** The correlation between predicted function capacity of lung micro‐ biome and lung lesions.
