# Supplementary figures and images for: Microbial communities in swine lungs and their association with lung lesions
Source: Microb Biotechnol. 2018 Dec 17;12(2):289–304. doi: 10.1111/1751-7915.13353 (PMC6389860; doi:10.1111/1751-7915.13353)

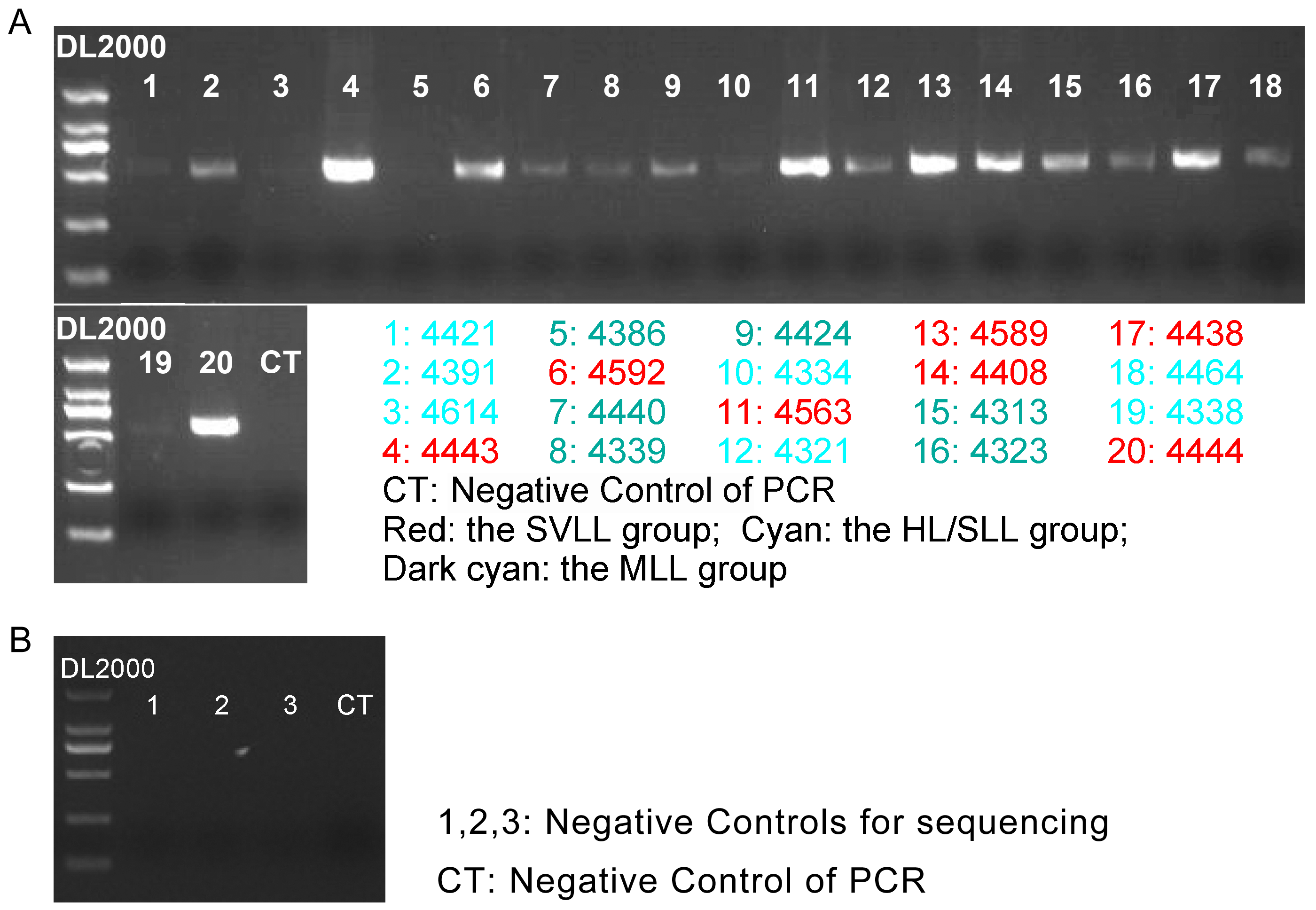

Supplement: Supplementary file 1 — Fig. S1. PCR results of 16S rRNA V3‐V4 region for 20 bronchoalveolar lavage fluid samples and 3 negative controls. [file MBT2-12-289-s001.tif]

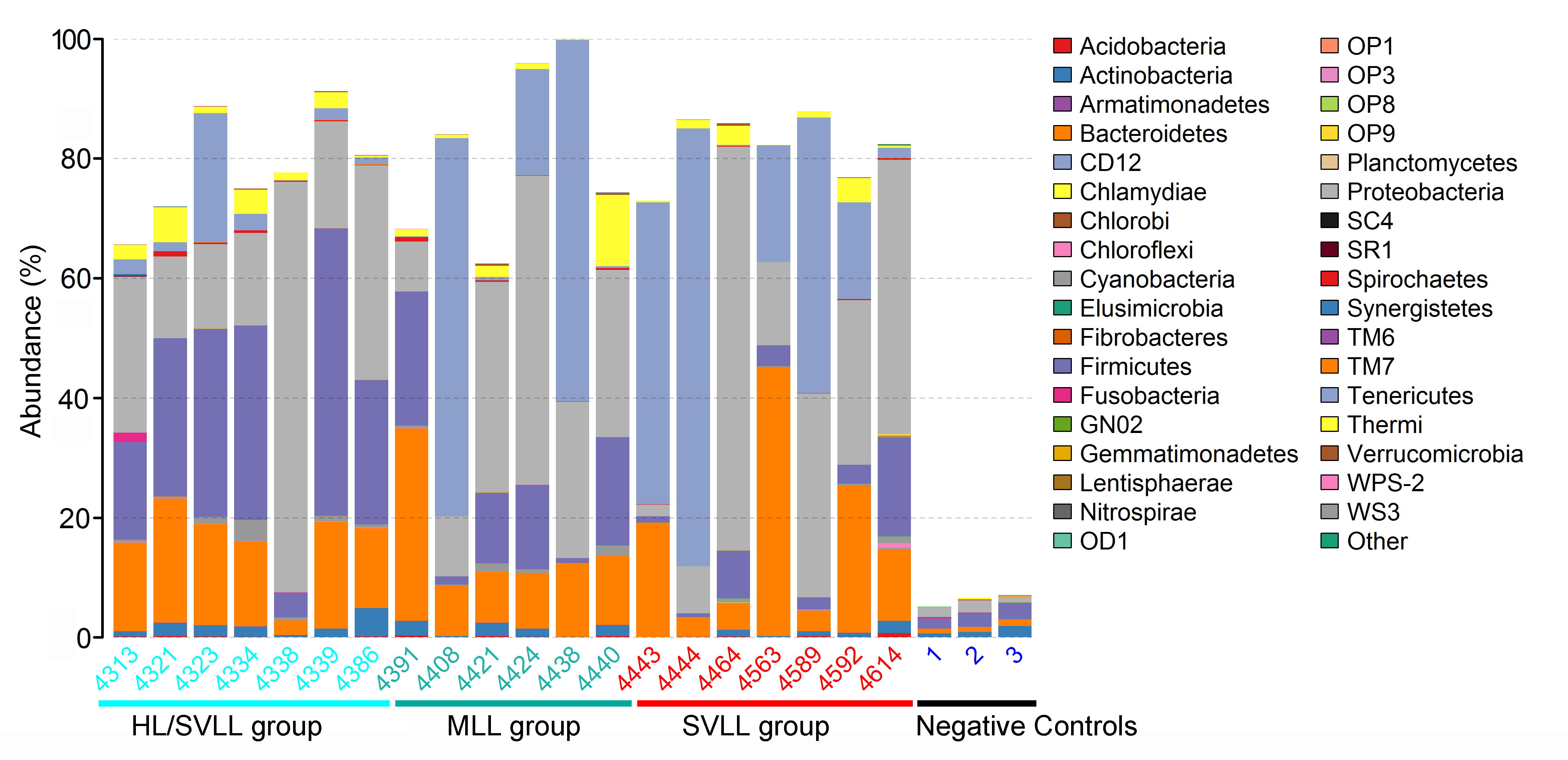

Supplement: Supplementary file 2 — Fig. S2. Microbial communities at the phylum level in 20 bronchoalveolar lavage fluid samples and three negative controls. [file MBT2-12-289-s002.tif]

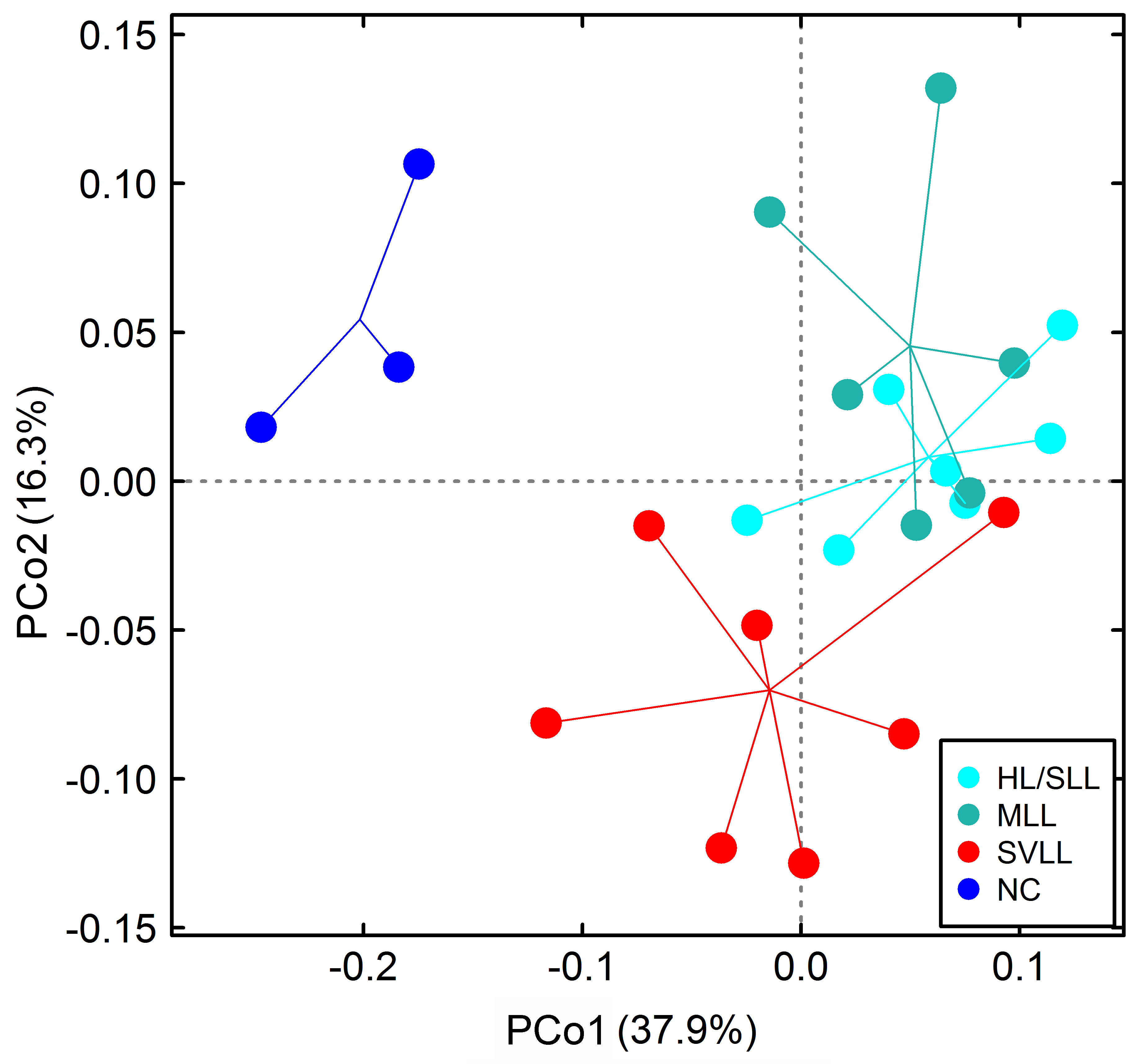

Supplement: Supplementary file 3 — Fig. S3. The principal component analysis for 20 bronchoalveolar lavage fluid samples and three negative controls based on the weighted UniFrac distances. [file MBT2-12-289-s003.tif]

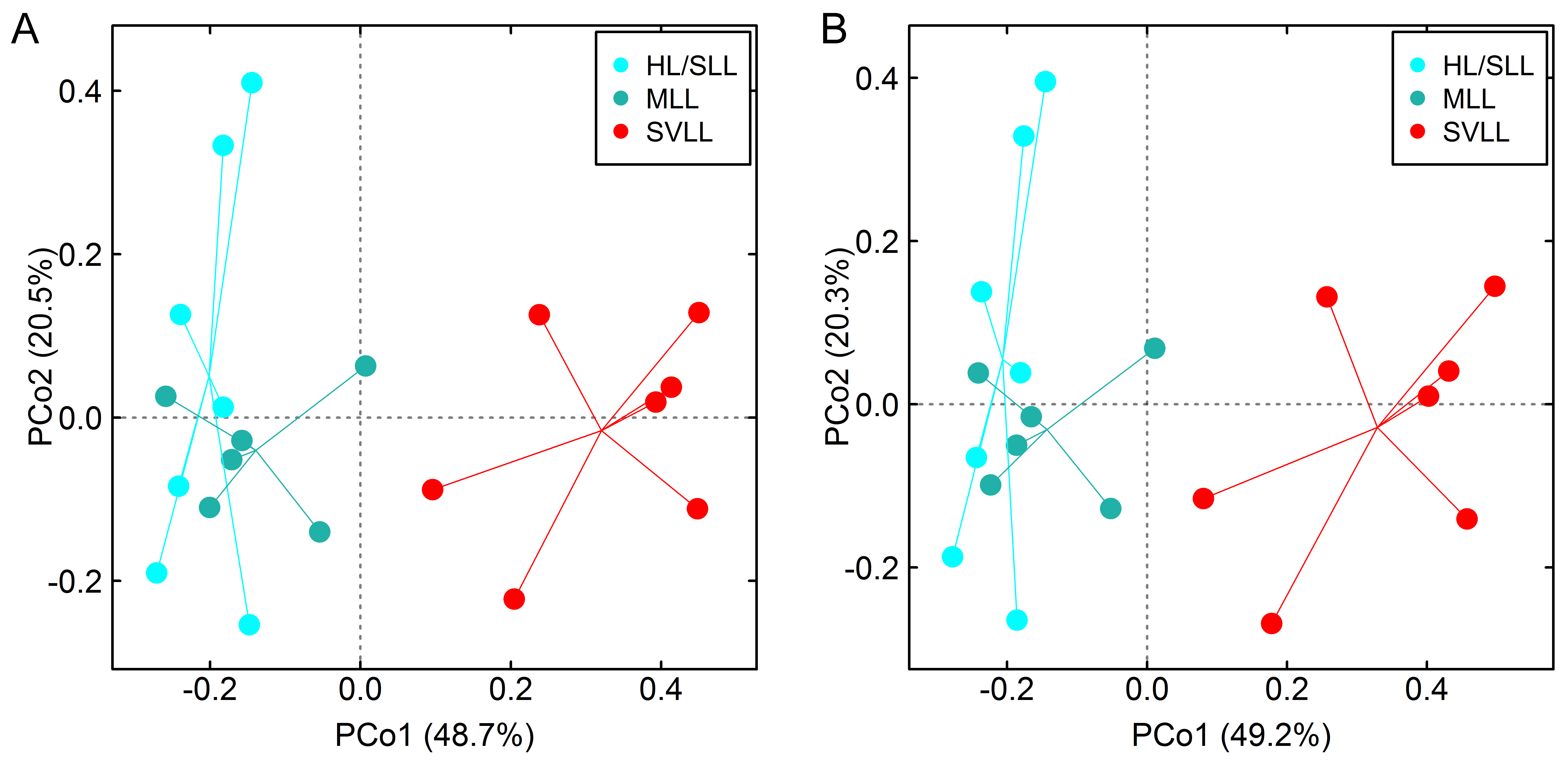

Supplement: Supplementary file 4 — Fig. S4. The principal component analyses for original OTUs of lung microbiota versus the new set of OTUs removing 21 OTUs shared by negative controls. [file MBT2-12-289-s004.tif]

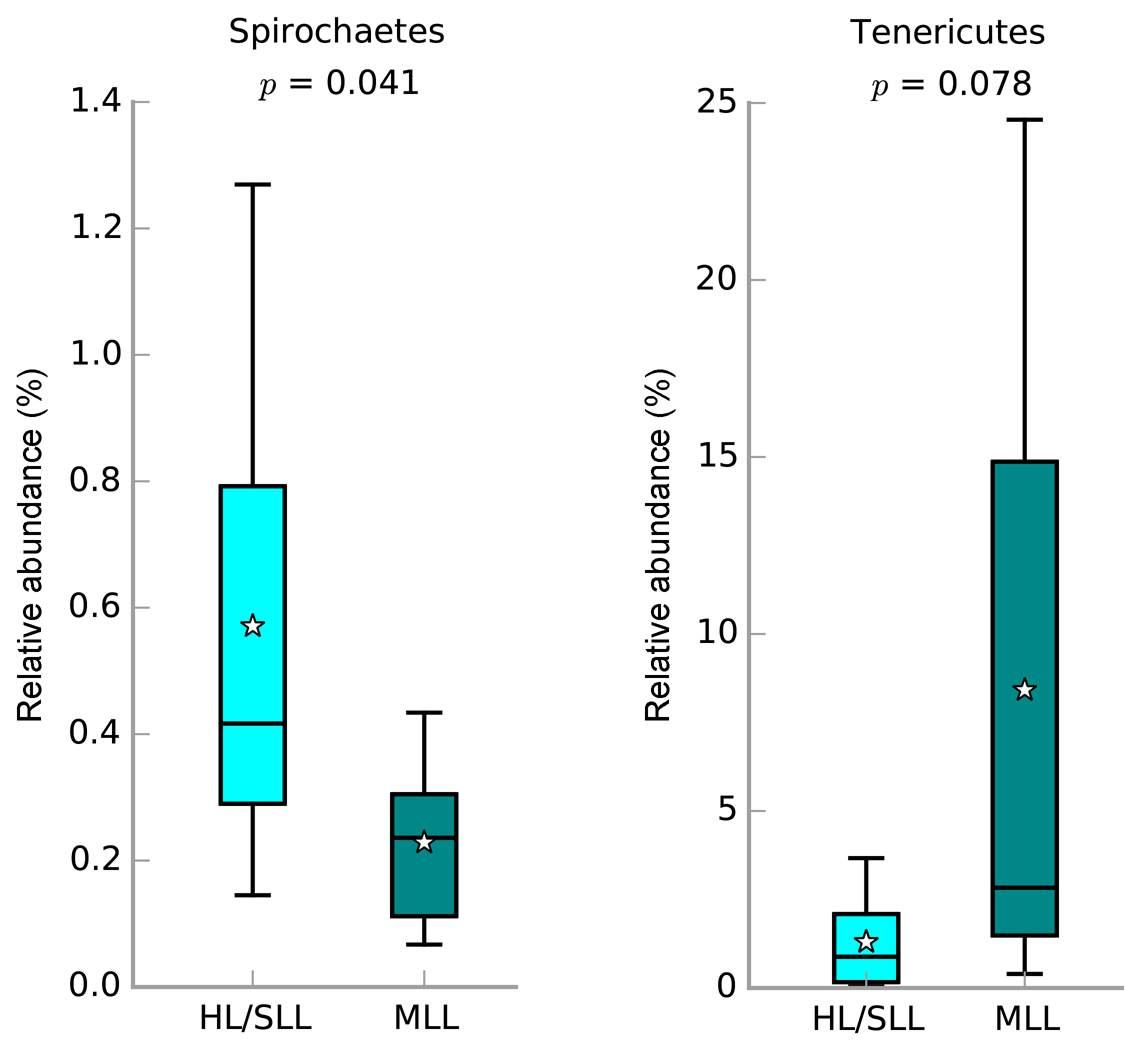

Supplement: Supplementary file 5 — Fig. S5. Comparison of Spirochaetes and Tenericutes between the HL/SLL group and the MLL group. [file MBT2-12-289-s005.tif]

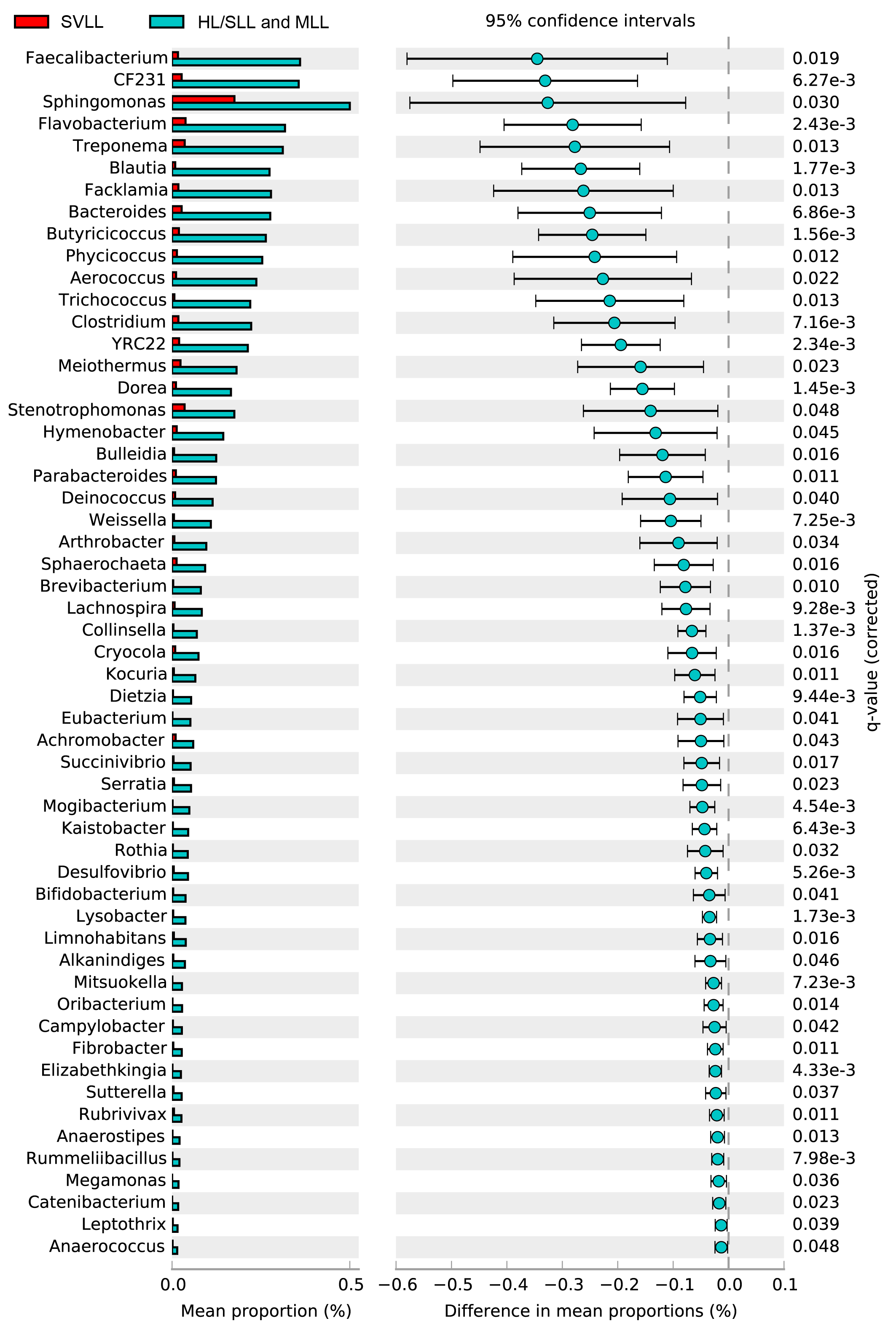

Supplement: Supplementary file 6 — Fig. S6. Comparison of lung bacterial genera with low relative abundances of < 0.5%. [file MBT2-12-289-s006.tif]

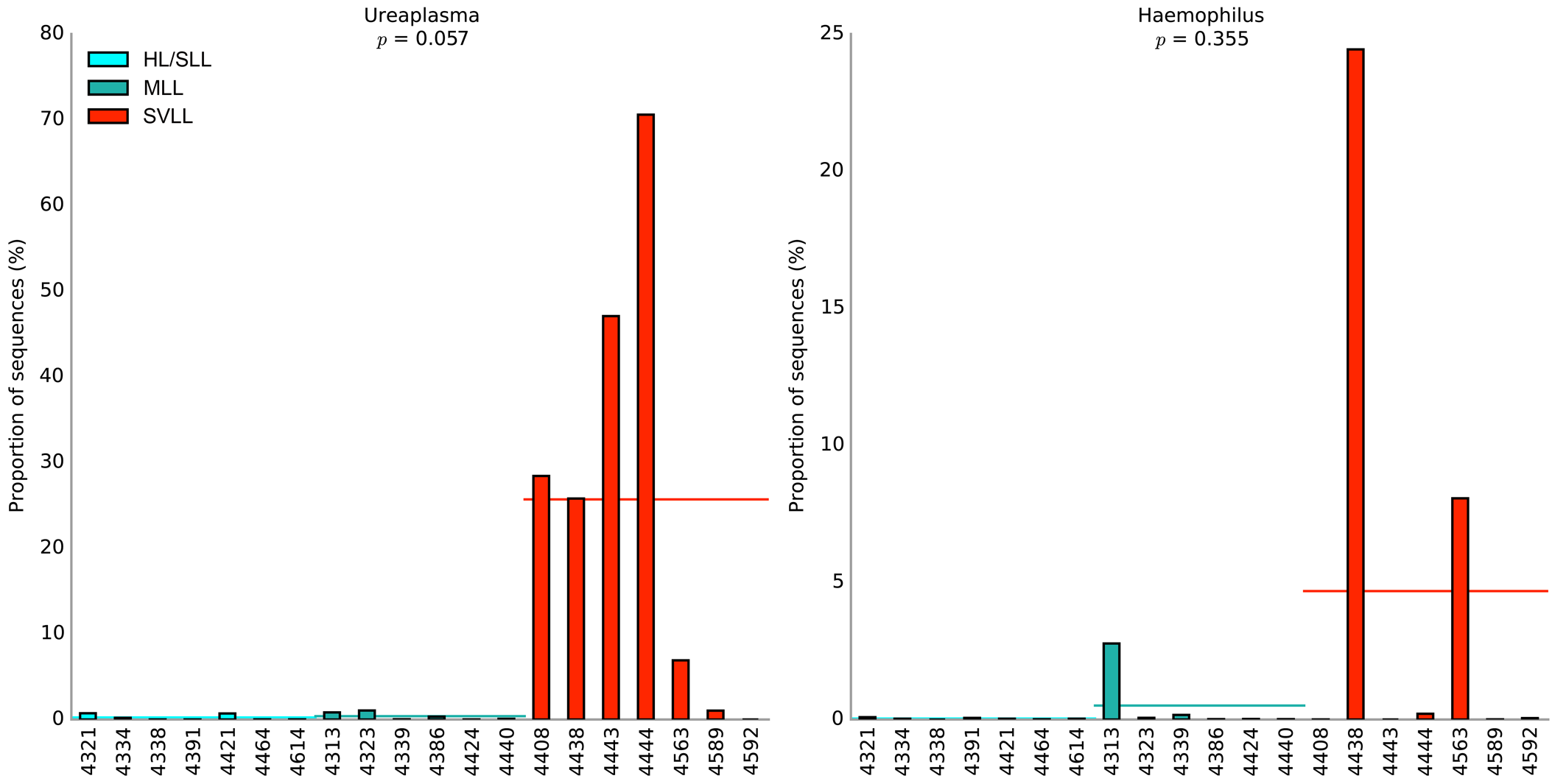

Supplement: Supplementary file 7 — Fig. S7. The relative abundance of Ureaplasma and Haemophilus. [file MBT2-12-289-s007.tif]
